# Fine Tuning RETFound with Clinically Guided Foveal ROI for Automated DRIL Classification in Diabetic Macular Edema OCT

**DOI:** 10.3390/diagnostics16111654

**Published:** 2026-05-27

**Authors:** Pavithra Kodiyalbail Chakrapani, Preetham Kumar, Sulatha Venkataraya Bhandary, Geetha Maiya, Shailaja Shenoy, Steven Fernandes

**Affiliations:** 1 Manipal Institute of Technology, Manipal Academy of Higher Education, Manipal 576104, India; pavithraforu@gmail.com (P.K.C.); geetha.maiya@manipal.edu (G.M.); 2 Department of Ophthalmology, Kasturba Medical College, Manipal Academy of Higher Education, Manipal 576104, India; sulatha.bhandary@manipal.edu (S.V.B.); shailaja.s@manipal.edu (S.S.); 3 Department of Computer Science, Design and Journalism, Creighton University, Omaha, NE 68187, USA

**Keywords:** disorganization of retinal inner layers, diabetic macular edema, optical coherence tomography, deep learning, disease, diabetes, health, transfer learing, biomarker, vision transformer

## Abstract

**Background/Objectives**: Disorganization of retinal inner layers (DRIL) is an important and supportive biomarker in optical coherence tomography (OCT) imaging for diagnosing the extent of diabetic macular edema (DME) in patients and anticipating visual outcomes. But the manual DRIL identification is subject to interobserver bias and requires a lot of time and effort from the experts. This research presents a novel, computerized, and clinically guided approach for the classification of DRIL that leverages the central 1 mm foveal region extracted through the annotations provided by the expert ophthalmologists and investigates the effectiveness of a transformer and Masked Auto Encoder (MAE) based foundation model (RETFound) as the primary approach. **Methods**: We fine-tuned and validated the RETFound model, utilizing accurate foveal center coordinates provided by the experienced ophthalmologists. Our approach emphasizes the macular region that is significant diagnostically, where DME biomarkers manifest more predominantly. To guarantee robust evaluation, the dataset was divided into 85% training and 15% held-out test sets. We performed 5-fold cross-validation exclusively on the training dataset with baseline, conservative, and moderate fine-tuning strategies, and the final model was evaluated on the independent, unseen test set. Convolutional neural network (CNN)-based transfer learning (TL) models (MobileNetV2, EfficientNetB0, InceptionV3, DenseNet121, and DenseNet169) were also assessed for comparative evaluation. **Results**: The RETFound model yielded the best outcomes under the conservative fine-tuning strategy, achieving a mean test accuracy (AC) of 0.9339 ± 0.0036 and an area under the curve (AUC) of 0.9660 ± 0.0028 on the independent held-out test set across the five fold-trained models. The moderate and baseline evaluations achieved comparatively lower outcomes, highlighting the effectiveness of the conservative approach. The RETFound model consistently outperformed CNN models, exhibiting stability and superior generalization for DRIL classification. We performed statistical validation using the Wilcoxon signed-rank test and 95% confidence intervals to confirm the robustness of the proposed method, and an ablation analysis showed that the fovea-centered region of interest (ROI) guidance consistently improved results when compared with whole OCT analysis. **Conclusions**: This research demonstrates that the deep-learning (DL) methods assisted by expert clinical knowledge with an anatomically aligned ROI could provide remarkable results in DRIL detection applications. This work attempts to establish an anatomically relevant framework for computerized DRIL identification that focuses on the highly crucial macular region, possibly helping in faster intervention and improved diagnosis in the management of DME.

## 1. Introduction

Diabetes mellitus is a widespread metabolic condition that impacts various organs, including the heart, kidneys, and eyes. Recurrent eyelid infections, early cataract formation, cranial nerve palsies, and, most critically, diabetic retinopathy (DR) are the ophthalmic complications of diabetes [1]. Severe hyperglycemia disrupts the retinal microvasculature, leading to capillary occlusion and increased vascular permeability. DME is the result of fluid leaking from blood vessels and building up in the macular region [2]. According to the 2025 International Diabetes Federation (IDF) Diabetes Atlas, 1 in 9 individuals aged 20–79, which is 11.1% of the population worldwide, has diabetes, while over 40% are clueless that they have acquired the condition. Further, the projections made by the IDF estimate that by 2050, the prevalence will rise by 46%, affecting nearly 853 million people, or approximately one in eight senior citizens [3].

### 1.1. Domain Aspects of DRIL

**Figure 1:**
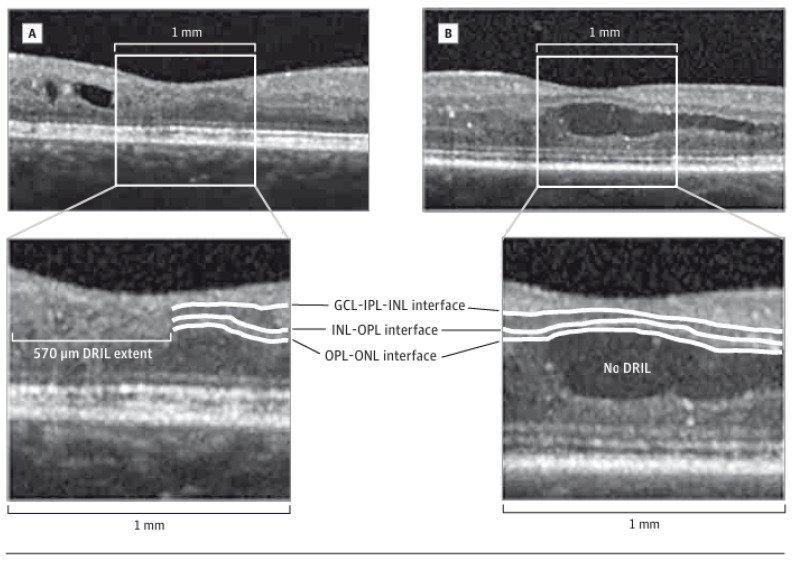
(**A**) The right side of the 1-mm box shows the partially visible margins of the retinal layers due to DRIL. (**B**) The 1-mm box indicates no DRIL and marks the border of each retinal layer [4].

DRIL, which can be estimated numerically and qualitatively employing OCT, is one of the crucial OCT imaging biomarkers linked to DME. In center-involved DME patients, both with ongoing and resolved DME, DRIL acts as a surrogate marker that is associated with visual acuity (VA). “Disorganization of the retinal inner layers was defined as the horizontal extent in microns for which any boundaries between the ganglion cell–inner plexiform layer complex, inner nuclear layer, and outer plexiform layer could not be identified” [4]. Figure 1 shows the presence and absence of the biomarker DRIL on OCT images.

### 1.2. Motivation

DME continues to rise along with the diabetes epidemic worldwide by affecting approximately 21 million people globally. Identification of DME at an early stage and severity characterization are important for timely diagnosis and treatment planning to prevent irreversible vision loss. The monitoring and diagnosis of eye diseases has been revolutionized by the advent of OCT, by offering very high-resolution images of the retinal tissues. DRIL is a vital imaging biomarker that has been predicted to be an accurate prognostic indicator. Several reports and longitudinal studies have illustrated that the extent and presence of DRIL are highly correlated with the baseline VA and are useful in predicting visual outcomes in the case of anti-VEGF treatment. Despite the morphological improvements, patients with severe DRIL show low rates of vision improvement, stating that DRIL evaluation is essential for therapeutic choices and counselling patients.

CNNs, in particular, have illustrated remarkable results in medical image processing. In ophthalmology, DL techniques have exhibited expert-level performance in several OCT-based disease screening tasks like age-related macular degeneration (AMD) classification, DR screening, etc. While working with limited medical datasets, TL has shown very good results with fine-tuning models for medical image classification.

### 1.3. Challenges in DRIL Detection

At present, clinicians identify DRIL manually with their expertise, typically focusing on the central 1 mm foveal region, since the intact structure of it contributes to accurate visual functioning. There are various issues involved in the identification of DRIL, including the time needed for thorough evaluation in busy clinical settings, inter-observer variability, and the huge amount of OCT images that are being acquired daily. The outlining of DRIL becomes much more challenging when multiple abnormalities or lesions coexist within the OCT images. These difficulties highlight the critical need for accurate, automated DRIL detection methods that can help medical professionals make timely and precise treatment decisions.

### 1.4. Novel Contributions of This Research

This research proposes a clinically guided approach for computerized DRIL classification that bridges the gap between artificial intelligence and clinical expertise. Our key contributions include the following:

- We propose a clinically guided DRIL classification architecture in OCT images that leverages the RETFound [5] foundation model, based on vision transformers and masked autoencoders for robust feature learning.
- We systematically evaluate baseline, conservative, and moderate finetuning strategies with RETFound. A rigorous evaluation protocol is employed using 5-fold cross-validation exclusively on the training set (85%) and a separately held-out test set (15%) for unbiased performance assessment. Additionally, statistical validation using Wilcoxon signed-rank testing and 95% confidence intervals is performed to confirm the robustness and consistency of the obtained results.
- Our approach concentrates on the exact region where DRIL has the strongest correlation with VA by cropping and assessing the central 1 mm foveal region, leading to better clinical interpretability and model performance. Further, to quantitatively validate the advantage of anatomically guided ROI selection, an ablation study comparing fovea-centered ROI guidance with whole OCT image evaluation is performed.
- We benchmark RETFound across five state-of-the-art CNN architectures (MobileNetV2, EfficientNetB0, InceptionV3, DenseNet121, and DenseNet169) to compare performance differences across diverse architectures.
- This research attempts to provide a qualitative visualization of self-attention maps of OCT images to help us understand the significant areas in the image that contribute the most to the decision-making process of the model.
- We make use of accurately annotated foveal center coordinates that are provided in the form of .json files, which are manually annotated by two certified and experienced ophthalmologists. This ensures the reliability of the annotations as well as the trained models, as we focus on the exact anatomical area of clinical interest, in the same way expert clinicians assess DRIL in practice.

The remainder of this paper is structured as follows: Section 2 provides a detailed explanation of the related work in DRIL detection in the past. Section 3 presents the novel methodology that includes the extraction of the central foveal region, data preprocessing, models, and training details. Section 4 describes the results of our experiments, including performance metrics across all evaluated models and fine-tuning approaches. Section 5 discusses the clinical use and impact of our results, discusses the limitations and contributions of our work, and outlines future research possibilities. Section 6 concludes the research and summarizes the results and contributions to automated DRIL assessment in DME.

## 2. Related Work

DRIL has emerged as a very useful OCT imaging biomarker that is crucial in assessing visual function. DRIL has been investigated by many researchers in the past for understanding its consequences and association with disease progression. To assess the prognostic usefulness of DRIL for VA changes in center-involved DME, Sun et al. carried out a pioneering longitudinal population analysis [4]. They discovered an intense association between baseline DRIL and alterations in DRIL over 4 months and 8-month VA outcomes. A line indicating loss in VA was correlated to a 250 μm increment in DRIL, whereas an improvement in VA was anticipated by a reduction in DRIL. DRIL has been demonstrated to serve as a reliable, noninvasive indicator for subsequent visual function in DME through this study. In patients with both in-progress and resolved DME, these authors investigated DRIL as an acceptable structural biomarker in further detail [6]. In particular, DRIL alterations at 4 months were better than other fundamental OCT measures, such as central subfield thickness (CST), at anticipating visual outcomes at 1 year. The study’s emphasis on subjective OCT layer assessment restricted its potential for computerized implementation in healthcare settings, regardless of its clinical value. Radwan et al. [7] discovered that though prolonged DRIL was correlated to worse results, the correction of DRIL was gradually significantly correlated with improvements in VA in DME patients. The investigation highlighted the usefulness of DRIL as a prospective imaging biomarker; however, it used subjective grading approaches that are not realistic for use in clinical settings.

Acon and Wu [8] claimed that even though spectral-domain OCT has been widely used for DME diagnosis and reliable biomarkers are still being identified completely, ML can also provide treatment decisions based on multi-modal image processing. In a cross-sectional investigation by Das et al. [9] encompassing 102 eyes, a substantial correlation between DRIL and poor VA with higher severity of DR was reported. Additionally, they observed that DRIL was associated with both increased CST and outer retinal disruptions. However, scalability was restricted given manual processing, as causal reasoning was impacted by the study’s observational approach. A study by Joltikov et al. [10] probed into the detection of DRIL in diabetic patients who did not have DME. According to their research, DRIL has been linked to impaired retinal functioning, which impacts perimetry performance, sensitivity to contrast, and VA. The research highlighted the necessity for automated early detection tools by emphasizing DRIL as a possible early biomarker of neuroretinal dysfunction even before apparent retinopathy.

Regardless of VA or any evidence of edema, the study by Nakano et al. [11] showed that the only OCT parameter substantially correlated to the extent of the metamorphopsia proved to be the length of DRIL. Although the study had its limitations with a small number of samples and lacked computerization, it uncovered an entirely novel aspect of DRIL’s clinical effectiveness. Nadri et al. [12] evaluated the association across DRIL, thinning of retinal nerve fiber layer (RNFL), disruption of ellipsoid zone (EZ) in DR. The experiment, comprising 104 subjects with different stages of DR, exhibited a significant correlation between the presence of DRIL, a rise in CST, evident EZ disruption, and lower RNFL thickness. The research team determined that DRIL functions as a credible morphological OCT biomarker for the degree of DR and visual loss. The research was cross-sectional, employing subjective OCT layer identification and providing no computerized DRIL detection approaches.

A comprehensive evaluation of seven significant investigations assessing DRIL as a biomarker in DME was carried out by Di-Luciano et al. [13]. The review supported the prognostic impact of DRIL by validating an elevated association between the magnitude of DRIL and decreased VA across several cohorts. It emphasized that the existence or lack of DRIL following therapy can signify potential vision improvement or vision deterioration. The review acknowledged, however, that clinical applicability was limited because all included trials used subjective OCT grading, lacked uniform DRIL measurements, and did not employ DL-based techniques. Singuri et al. [14] reported the fact that DRIL evaluated on OCT images has a strong association with advances in DR severity and poor visual acuity, highlighting DRIL as a prognostic biomarker with high clinical relevance. To automatically recognize DRIL in OCT B-scans, Singh et al. [15] experimented with four CNNs based on DL. The best model demonstrated high sensitivity and specificity, attaining an accuracy of 88.3% on a dataset making up more than 5992 images. Although this research was among the initial attempts to computerize DRIL identification, it lacked explainability. In accordance with the standards given in the literature, DRIL, which is more pronounced and results in vision loss, is identified in the central 1 mm region of the retina. Without paying attention to the central 1 mm region of the retina, the authors evaluated OCT images labeled as normal or DRIL.

Tripathi et al. [16] introduced a fuzzy logic-based method for assessing the severity of DME by identifying biomarkers such as cystoids, hyperreflective retinal foci (HRF), and DRIL using OCT scans. The model attained an accuracy of 93.3% in DRIL identification. The fuzzy rule-driven system, although hopeful, proved inefficient in scalability and adaptability compared with DL strategies. Toto et al. [17] recently published an innovative DL pipeline incorporating models such as RegNetX, ConvNeXt, and Yolov7 for the simultaneous recognition of DRIL and hard exudates. For DRIL classification, their ensemble algorithm’s accuracy, sensitivity, and specificity were 91.1%. Despite the complicated nature of the model, making adoption a challenge, the ensemble technique demonstrated resilience. The central 1 mm region of the OCT scan was not the main focus of the ensemble model, but the presence of DRIL in this region is concerning. With the objective to explore OCT biomarkers, such as DRIL, as indicators of anti-VEGF drug effectiveness in DME individuals, Ruiz-Medrano et al. [18] performed a clinical investigation. One of the factors that was considerably tied to the requirement of shifting to dexamethasone implants during treatment was the existence of DRIL. Although this study does not use an automated identification strategy, it emphasizes the therapeutic significance of early DRIL detection for individualized treatment scheduling. Recently, Kodialbail Chakrapani et al. [19] (our prior work) have presented a self-supervised learning approach that uses whole-image processing and a spatially aware Bootstrap Your Own Latent (BYOL) architecture for automated DRIL classification from OCT B-scans. The usefulness of domain-specific self-supervised representation learning for DRIL identification was demonstrated by the suggested framework’s classification accuracy (99.39%) on a separate test set. However, without a clear anatomical direction, the method depended on convolutional backbones processing the whole OCT data. As shown in Table 1a, all the clinical and observational studies report inter-observer agreement metrics, and DL-based DRIL classification study results are listed in Table 1b.

Most of the publications have reported the results of clinical trials that focus on understanding the disease progression with DRIL and its association with all other biomarkers. Those studies that have attempted DRIL identification using DL models do not focus on clinically relevant ROI, but they make use of whole B-scans. They lack the integration of expert clinical knowledge with DL-based model architectures for DRIL detection. Limited systematic assessment of different pretrained models for localized retinal evaluation and a lack of rigorous fine-tuning and cross-validation methods for DRIL detection have led to the implementation of the proposed methodology.

## 3. Materials and Methods

### 3.1. Dataset

The private, anonymous, original, fovea-centered OCT dataset utilized for the research has been acquired from the Department of Ophthalmology at Kasturba Medical College (KMC), Manipal, MAHE, Manipal. With authorization code IEC1-287/2022, this study has been approved by the Kasturba Medical College and Kasturba Hospital Institutional Ethics Committee. This research was conducted in compliance with the Declaration of Helsinki’s principles and the ethical guidelines stated by the Kasturba Medical College and Kasturba Hospital Institutional Ethics Committee. Excellent-quality, horizontal, foveal-centered raster scans of DME patients were retrospectively gathered. These OCT images were collected around January 2019 and August 2022. As part of a comprehensive DRIL classification technique, the OCT pictures collected from KMC were obtained using certified Zeiss Cirrus HD OCT 5000 (Carl Zeiss Meditec AG, Jena, Germany) imaging equipment. The study takes into consideration 823 (original resolution of 938 × 625 pixels) fovea-centered OCT B-scans, 429 of which exhibit DRIL and 394 scans that do not possess DRIL. All these 823 OCT B-scans were collected from 823 unique patients (823 eyes), where an OCT volume per patient consisted of 5 raster scans, and the B-scan (signal strength greater than 7) passing through the central foveal region is considered for the research. With this design, it is ensured that no two scans of the same patient or eye are used in the research, thereby avoiding the risk of data leakage. Table 2 provides detailed characteristic information related to the KMC DRIL OCT dataset.

### 3.2. OCT Dataset Annotation Protocol

The recommended approach involves the use of expert-verified consensus annotations compiled by two senior ophthalmologists from the reputable medical facility Kasturba Medical College, Manipal, India. DME is one of the diabetic retinal conditions that each ophthalmologist has identified and addressed during their more than 23 years of professional experience. Marking the foveal centers of OCT scans and labeling them for DRIL or No-DRIL was independently performed by the two ophthalmologists. They used a keypoint labeling software called LabelImg [20] to identify the exact foveal center of an OCT B-scan in addition to DRIL labels. Each OCT image’s foveal center’s *x*-*y* pixel coordinates have been recorded in the annotations, which were saved in the .json format. Foveal center annotations for model development were finalized by a consensus evaluation. High trustworthiness, clinical correctness, and clinical relevance have been ensured by the unbiased labeling of the annotations and their eventual reconciliation to create a consensus ground truth. By eliminating annotation bias and strengthening model generalizability, this provides a strong basis for DL model training and validation.

### 3.3. Inter-Observer Variability Statistics

**Figure 2:**
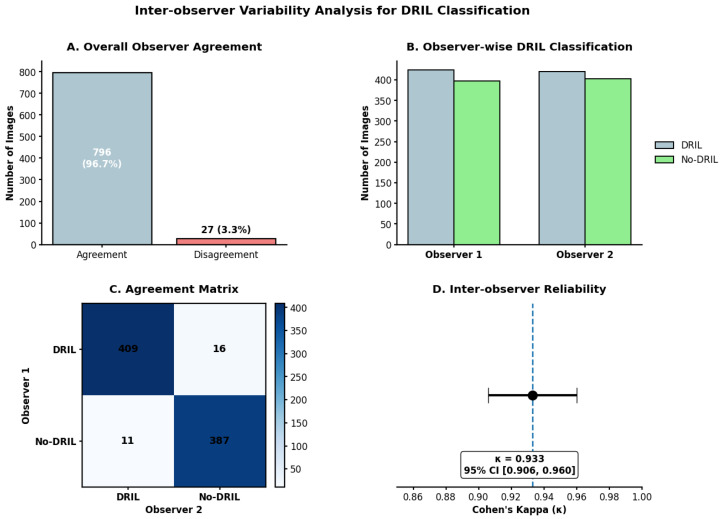
Inter-observer variability analysis for DRIL classification in OCT images. (**A**) Overall agreement between the two observers across all the 823 scans, with 796 cases (96.7%) classified concordantly. (**B**) Distribution of DRIL-present (DRIL) and DRIL-absent (No-DRIL) labels assigned by each observer individually. (**C**) Agreement matrix summarizing concordant (true positive: 409; true negative: 387) and discordant (false positive: 16; false negative: 11) categories. (**D**) Cohen’s kappa coefficient (κ = 0.933, 95% CI [0.906, 0.960]), indicating almost perfect inter-observer reliability, where the dashed vertical line represents the estimated Cohen’s kappa value.

The interobserver agreement for DRIL present and DRIL absent annotations was evaluated using Cohen’s kappa coefficient. The standard error of the kappa statistics was computed to estimate the 95% confidence intervals as

$$SE\left(\kappa \right)=\sqrt{\frac{p_{o}\left(1-p_{o}\right)}{N{\left(1-p_{e}\right)}^{2}}}$$

where $p_{o}$ indicates the observed ratio of agreement, $p_{e}$ denotes the agreement expected by chance, and *N* is the total number of B-scans that were evaluated. A threshold of p<0.05 was used to assess the statistical significance. A total of 823 B scans were analyzed, and the two observers arrived at an overall agreement of 96.7% (796/823 scans), with 27 cases of disagreement (3.3%). The Cohen’s kappa value of κ=0.933 (95% CI: 0.906–0.960) demonstrates an excellent level of interobserver reliability for DRIL annotations. In the confusion matrix, shown in Panel 3 of Figure 2, both observers agreed that 387 B scans did not possess DRIL and could concordantly detect DRIL in 409 scans. Table 3 provides the inter-observer statistics of the DRIL dataset.

Observer 2 reported DRIL in 11 of the 27 discordant OCT scans that Observer 1 considered No-DRIL, while Observer 1 discovered DRIL in 16 scans that Observer 2 thought were No-DRIL. The final dataset, after the consensus annotation protocol, included 394 No-DRIL images (47.9%) and 429 DRIL images (52.1%). To ensure anatomical accuracy and consistency among the annotations, the two observers employed independent labeling of OCT B-scans for DRIL identification and a collaborative agreement between them for the localization of the fovea through center identification. This research focuses more on anatomical or clinical accuracy than annotation reproducibility. We followed the consensus to integrate the judgment of doctors. For DRIL and No-DRIL labeling, among the 823 images, there were 27 discordant cases, which were later resolved through discussion in a collaborative manner. But for foveal center annotations, the experts followed a collaborative discussion approach, as the accurate identification of the fovea is crucial for characterizing the disease, and because pathological retinas include minor anatomical changes. Typically, a normal retinal OCT scan shows a concave dip in the retinal contour, a slight swelling of the EZ, and an absence of inner retinal layers, which differentiate them from pathological retinal structures. It is challenging to identify the foveal center even for experienced doctors in the case of DME, as the lesions can alter the morphology significantly. Few standards have been put forth to determine the foveal centers to handle this complexity and ensure consistent labeling. For foveal localization in the case of diseased retinas, observers together attempted to identify the point of minimal RNFL thickness as the fovea center [21,22].

### 3.4. Experiments

The methodology adopted for the proposed research comprises four distinct steps, which include the following: preprocessing the original OCT B-scans and .json files and extraction of the foveal region; preparation of the dataset and stratified splitting; training the RETFound model with baseline, conservative, and moderate fine-tuning techniques and detailed evaluation of the best model on the held-out test set. The proposed method uses the statistical measures AC, sensitivity (SE), specificity (SP), AUC, and Matthews correlation coefficient (MCC) for the model evaluation. The entire pipeline is aimed at utilizing expert annotations for anatomically accurate DRIL classification, along with systematic assessment of five state-of-the-art TL architectures. Algorithm 1 demonstrates the pseudocode for the preparation of OCT data and model training-evaluation procedures.
**Algorithm 1:** RETFound-Based DRIL Classification Architecture.1:**Input:** OCT images with foveal annotations2:**Model:** RETFound (Vision Transformer)3:// Phase 1: Preprocessing the OCT Images and extracting central foveal 1 mm region4:**for all** class c∈{DRIL,No-DRIL}
**do**5:    **for all** image $I_{i}$ in class *c*
**do**6:        Load *I_i_* and annotation file7:        Read foveal center coordinates $f_{x}$8:        $I_{crop}\leftarrow \mathrm{CropCentral}\left(I_{i},f_{x},\mathrm{width}=1\;\mathrm{mm}\right)$9:        $I_{enhanced}\leftarrow \mathrm{CLAHE}\left(I_{crop}\right)$10:        Save $I_{enhanced}$11:    **end for**12:**end for**13:// Phase 2: Splitting the dataset for training and testing14:D← Read all preprocessed images15:Shuffle D with seed = 4216:$D_{train},D_{test}\leftarrow \mathrm{Split}\left(D,0.85,0.15\right)$17:// Phase 3: Cross-validation and model training18:Initialize K=519:**for**    k=1 to *K* **do**20:    Split $D_{train}$ into Train and Validation sets21:    Initialize RETFound with pretrained weights22:    Freeze backbone layers23:    Train classification head24:    Unfreeze layers for fine-tuning25:    Validate on Validation set26:**end for**27:// Phase 4: Model selection and evaluation28:Select best model based on cross-validation performance29:Evaluate final fold-specific trained models on the held-out test set30:Compute-AC, SE, SP, AUC, MCC and Plot Visualizations and other curves

#### 3.4.1. Extraction of Foveal Region, Preprocessing and Stratified Splitting

The very initial phase aims at the extraction of the clinically crucial central 1 mm foveal region (which is approximately 156 pixels wide) from each OCT image, making use of the .json files as detailed in Algorithm 1 (lines 3–10). The cropped scans are then improved using contrast-limited adaptive histogram equalization (CLAHE) with a tile grid dimension of 8 × 8 and a clip threshold of 2.0 to increase contrast and highlight retinal layer attributes. After preprocessing, we use stratified dataset splitting and random shuffling (seed = 42) to split the dataset into training (85%) and test (15%) sets to ensure balanced class representation as depicted in the Algorithm 1 (lines 13–16). The splitting operation is expressed as follows:

$$D_{\mathrm{train}},D_{\mathrm{test}}=\mathrm{Split}\left(D,0.85,0.15\right).$$

To ensure reproducibility and to reduce bias during selection, we use the following approach for data splitting. The entire dataset is randomly shuffled (using a random seed = 42) to guarantee consistent splits across all the experimental runs. Given the potential for class imbalance in DRIL incidence, stratification is maintained to preserve class distribution across all subsets. The training split $D_{\mathrm{train}}$ has been used for model parameter learning with cross-validation. During the tuning of hyperparameters, the 15% validation set obtained from $D_{\mathrm{train}}$ provides independent result assessment, and the held-out test set ($D_{\mathrm{test}}$) is used for model evaluation. This approach guarantees that the proposed implementation aligns our method with the clinical diagnostic approaches by making sure that the future analysis focuses on the anatomically relevant foveal region, where DRIL has the strongest association with the visual function.

#### 3.4.2. Training RETFound Model with Cross Validation Followed by Progressive Finetuning

A ViT-based MAE model, RETFound, pretrained on the retinal OCT images, was leveraged, initially by freezing the encoder and training only the classification head using the AdamW optimizer (*lr* = 1 × 10^−3^, weight decay = 0.05), 30 epochs, and batch size = 16, with a warmup + cosine learning rate scheduler (5 warmup epochs). To enhance generalization by addressing class imbalance, the training included features such as extensive spatial augmentations, mixup augmentation (α=0.2), and label smoothing BCE loss (smoothing =0.1, $\text{pos\_weight}\approx \frac{\text{No-DRIL}}{\text{DRIL}}$), along with gradient clipping (max norm =1.0). We adopted two strategies for fine-tuning the RETFound model: conservative (top 4 blocks, $lr=5\times 10^{-6}$) and moderate (top 8 blocks, $lr=2\times 10^{-6}$). For each strategy, the model was trained for 40 epochs using different learning rates for the head and backbone. Final evaluation was performed on the independent held-out test set using test-time augmentation (including rotation and horizontal/vertical flipping), where each fold-trained model obtained through cross-validation was evaluated once on the same test set. Performance metrics, including AUC, accuracy, sensitivity, specificity, and MCC, were reported with optimal threshold values determined using Youden’s index along with various plots of the metrics.

#### 3.4.3. Pretrained CNN Implementation

Additionally, we have evaluated the baseline versions of 5 pretrained models for DRIL classification employing 5-fold cross-validation on the complete dataset of 823 OCT scans. Each of the five chosen TL models represents a distinct choice, helping us make more accurate decisions for DRIL classification:

- MobileNetV2—an efficient model that uses depthwise separable convolutions and inverted residual blocks, specially crafted for mobile and resource-cons trained environments.
- EfficientNetB0—a compound-scaled model that optimizes depth, width, and input resolution jointly, helping to achieve an optimal balance between classification performance and computational efficiency.
- InceptionV3—a multi-scale model that utilizes parallel convolutional filters of different-sized kernels to capture features across multiple receptive fields.
- DenseNet121—a model with dense connections where each layer receives feature maps from all preceding layers, allowing for feature reuse and improved gradient propagation.
- DenseNet169—a deeper version of DenseNet that enhances representational capacity through extra dense blocks.

Let M={MobileNetV2,EfficientNetB0,InceptionV3,DenseNet169,DenseNet121} represent the set of models that are evaluated. For every model M∈M, we use TL by initializing the network with pre-trained ImageNet weights. To preserve the learned low-level and mid-level feature representations, all convolutional layers in $M_{\mathrm{base}}$ are initially frozen. A new classification head, tailored for binary DRIL classification, is then appended to the pre-trained base network. The classification head is defined as

Head=GlobalAvgPool→Dropout(p=0.3)→Dense(1,sigmoid).

The spatial dimensions are reduced with the global average pooling layer while preserving channel-wise information. A dropout layer with a dropout ratio of 0.3 helps in regularization to prevent overfitting. The final output of positive class probabilities is provided by the final dense layer with sigmoid activation. Five-fold cross-validation has been employed to guarantee robust performance and improve the model’s generalization. For each fold f∈{1,2,3,4,5}, the complete dataset of 823 OCT images is divided into training subsets that are fold-specific $D_{\mathrm{train}}^{\left(f\right)}$ and followed by a validation subset $D_{\mathrm{val}}^{\left(f\right)}$. With each fold, the model $M_{f}$ is trained on the fold-specific training subset $D_{\mathrm{train}}^{\left(f\right)}$ with the frozen base, binary cross entropy loss for 10 epochs using the Adam optimizer. Once the model is trained, predictions are generated on the validation set as

$${\hat{y}}_{\mathrm{val}}^{\left(f\right)}=M_{f}.\mathrm{predict}\;\left(D_{\mathrm{val}}^{\left(f\right)}\right).$$

After the execution of every fold, we compute performance metrics, including AC, sensitivity (SE), specificity (SP), AUC, and Matthews correlation coefficient (MCC). Once all the cross-validation fold executions are completed, the mean ($\mu_{M}$) and standard deviation ($\sigma_{M}$) of each metric are computed to quantify the consistency of results and model robustness.

#### 3.4.4. Implementation Details

RETFound experiments were conducted using PyTorch (version 2.9.1) on a high-performance computing (HPC) platform featuring NVIDIA A100 80 GB PCIe GPUs with CUDA 12.8. To meet the input requirements of the RETFound Vision Transformer, input OCT images were scaled to 224 × 224 pixels and transformed to 3-channel RGB format. CNN model executions were performed with Google Colab with GPU acceleration and the TensorFlow 2.x framework in a Python 3.x environment. The entire training and evaluation pipeline was implemented under these configurations. Model-specific input dimensions were determined according to the default specifications of each architecture: DenseNet121, DenseNet169, MobileNetV2, and EfficientNetB0 used input images of size 224×224 pixels, whereas InceptionV3 required 299×299 pixels, in accordance with standard TL requirements.

## 4. Results

Table 4 demonstrates the mean results of five-fold cross-validation performed on the training data using the RETFound model. When analyzing the results of all three strategies, baseline, conservative, and moderate appear consistent and strong. Among the three strategies, the conservative method yielded the highest mean performance during the cross-validation. The conservative strategy achieved the highest AUC of 0.9971 ± 0.0016, along with improvements in AC (0.9733), SE (0.9564), SP (0.9920), and MCC (0.9476), exhibiting good discriminative ability and class balance when compared with the other two approaches. The moderate strategy also demonstrated comparable results; it did not outperform the conservative method, indicating that unfreezing a very few top blocks of the transformer yields an optimal trade-off between overfitting and feature learning. Table 5 displays the results of the held-out test set evaluation performed using the conservative approach. On the held-out test set, the conservative strategy demonstrated robust generalization performance, with an AUC of 0.9660 ± 0.0028 and AC of 0.9339 ± 0.0036, with balanced SP (0.9424 ± 0.0093) and SE (0.9262 ± 0.0129) across the five fold-trained models. Effective classification performance on unseen data is further confirmed by the obtained MCC of 0.8680. The overall findings confirm the efficacy and generalizability of the RETFound-based DRIL classification framework; however, a small reduction in performance relative to cross-validation results is expected due to stricter evaluation on completely unseen data.

Table 6 shows the mean values obtained for the performance measures during the 5-fold cross-validation of five different pretrained CNN models with the complete dataset of 823 images. The best-performing baseline models among the assessed pre-trained CNN architectures were EfficientNet B0 and DenseNet121. While DenseNet121 achieved the highest AC (0.9526±0.0203), SP (0.9847±0.0112), and MCC (0.9073±0.0388), indicating slightly better overall balanced classification performance, EfficientNetB0 demonstrated strong discriminative capability for identifying DRIL cases with the highest mean AUC (0.9875±0.0101) and SE (0.9238±0.0371). When compared with these state-of-the-art pre-trained CNN models, the RETFound framework consistently demonstrated superior performance across all evaluation measures. Notably, even the baseline RETFound model achieved a competitive AUC (0.9869), closely matching the best-performing CNN baselines, while the conservative fine-tuning strategy substantially outperformed all CNN models, attaining an AUC of 0.9971 and an accuracy of 0.9733. Further, better class-balanced results were yielded by RETFound with higher values for SE, SP, and MCC values, demonstrating the enhanced reliability in differentiating DRIL and No-DRIL cases, as opposed to CNN-based models.

**Figure 3:**
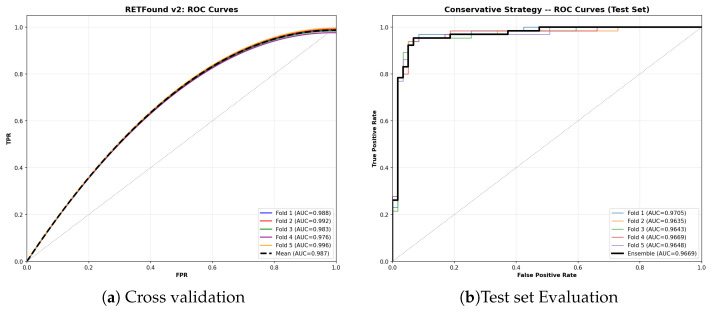
Receiver Operating Characteristic (ROC) curves for the RETFound model during the (**a**) 5-fold cross-validation and (**b**) Test set Evaluation based on Conservative Strategy.

**Figure 4:**
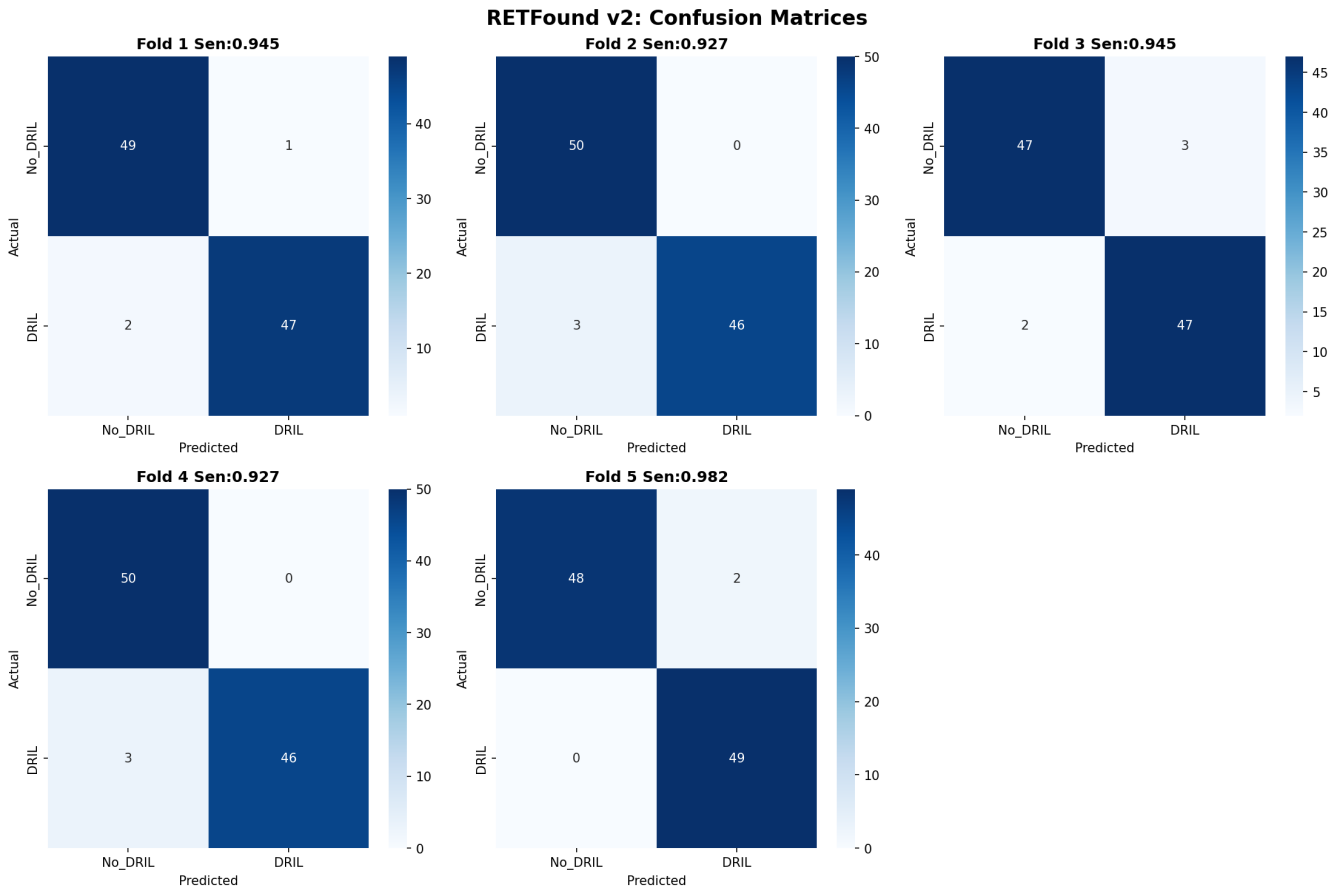
Fold-wise confusion matrices of the conservative fine-tuned RETFound model during 5-fold cross-validation on the 85% training subset. Each panel corresponds to one validation fold (Fold 1–Fold 5). Rows depict the ground-truth class labels (No-DRIL and DRIL), while columns depict the predicted class labels. The numerical values within each cell indicate the number of correctly and incorrectly classified OCT B-scans for the corresponding fold. Above each confusion matrix is the sensitivity (Sen) attained in each fold.

**Figure 5:**
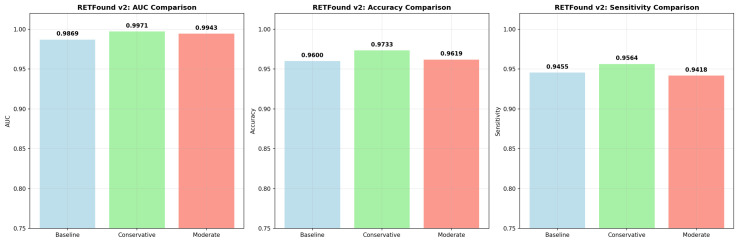
Comparison of baseline and two fine-tuning strategies (conservative and moderate) for the RETFound model applied to DRIL classification in OCT images, evaluated using 5-fold cross-validation.

**Figure 6:**
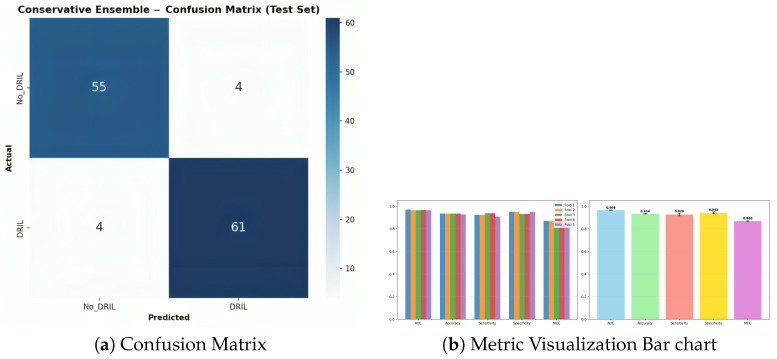
Plots generated during the test set evaluation of the conservative RETFound model. (**a**) Confusion Matrix (**b**) Visualization of Performance Metrices.

**Figure 7:**
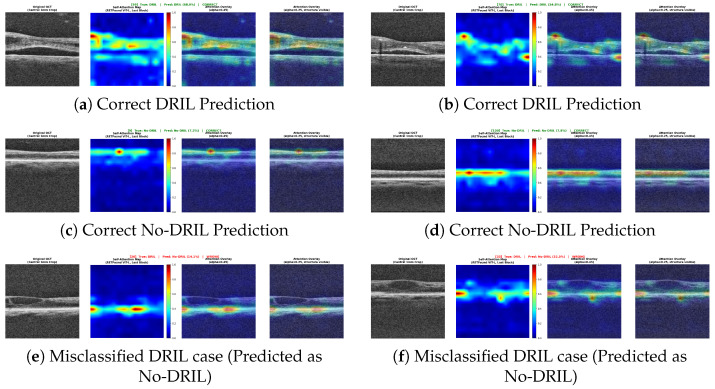
Qualitative analysis of self-attention maps generated by the conservative fine-tuned RETFound model. (**a**,**b**) Correctly classified DRIL cases show irregular and more dispersed attention patterns that correspond to disrupted retinal layers. (**c**,**d**) Correctly classified No-DRIL cases show attention concentrated along continuous retinal layers, indicating normal retinal layer structure. (**e**,**f**) Misclassified DRIL cases (predicted as No-DRIL) demonstrate diffuse or misaligned attention, suggesting that the model might focus on relatively preserved regions and overlook subtle DRIL features. These attention maps give qualitative insights into model behavior and should be interpreted as indicative rather than definitive explanations of model decisions.

Figure 3, Figure 4 and Figure 5 demonstrate the ROC curves, confusion matrices, and the finetuning strategy metric visualizations generated during the 5-fold cross-validation of the RETFound model, respectively. Figure 6 shows the confusion matrix and the bar chart visualizing various metric values on the test set evaluation of the conservative RETFound model. The RETFound model is a transformer-based model; it divides the 224 × 224 input OCT image into a grid of 14 × 14 = 196 small square patches, where each patch is 16 × 16 pixels. The model utilized a special token (classification token), which is responsible for the final DRIL and No-DRIL prediction. This token gathers information from the 196 image patches to make the decision. The model has multiple attention heads, and scores from all these heads were averaged to generate a single attention map. The final attention maps were resized and normalized for visualization. Red and yellow areas highlight which parts of the OCT image were most crucial to the model’s decision. The Figure 7 demonstrates the self-attention maps generated by the RETFound model on DRIL and No-DRIL images.

### 4.1. Statistical Significance Analysis

To assess the robustness of the observed performance differences, we have computed 95% confidence intervals using the standard errors across the 5 folds. We performed Wilcoxon signed-rank tests on the fold-wise results of both the conservative and moderate strategies to evaluate the statistical significance between the strategies. We utilized the foldwise AUC values with a significance level of *p* < 0.05. When compared with the moderate strategy (0.9943; 95% CI: 0.9913–0.9973) and baseline strategy of 0.9869 (95% CI: 0.9802–0.9936), the conservative fine-tuning strategy achieved a higher mean AUC (0.9971; 95% CI: 0.9958–0.9984). However, the Wilcoxon signed-rank test indicated that this difference was not statistically significant (*p* = 0.125). Statistical analysis indicates that both fine-tuning strategies yield consistently high results, with no statistically significant benefit of one over the other.

### 4.2. Comparison with Existing State-of-the-Art Methods

The results of the proposed RETFound-based model were compared with the previously published studies for DL-based DRIL OCT classification to further evaluate the effectiveness of the proposed framework as reported in Table 7. To present a fair comparison, the performance of the proposed method corresponds to the independent held-out test set, rather than cross-validation results. An important thing to note about our prior work, Kodiyalbail Chakrapani et al. [19], is that the two-stage self-supervised BYOL framework required pretraining on 108,309 unlabeled OCT images and approximately 50 h of backbone training. However, the proposed RETFound-based approach straightaway employs a domain-specific transformer-based foundation model along with ROI guidance and completes the entire pipeline of preprocessing, fine-tuning, cross-validation, held-out test evaluation, and interpretability using only 823 annotated OCT images in approximately 1.94 h, exhibiting substantially improved computational efficiency.

### 4.3. Key Observations

The RETFound model exhibited very good performance consistently across all evaluation settings, with the conservative strategy providing the best cross-validation outcomes in terms of AUC, accuracy, and MCC. RETFound yielded straightforward and clear enhancements when compared with pretrained CNNs, highlighting the effectiveness of transformer-based feature learning for OCT image classification. There was a slight reduction in the outcomes of the evaluation of the held-out test set, but the model maintained very good generalization abilities with balanced sensitivity and specificity. This implies that the model might effectively capture clinically relevant features without overfitting. Further, with several models exceeding the 0.92 accuracy level, often regarded as clinically acceptable for diagnostic support applications, these findings collectively show that the fovea-centric TL strategy provides state-of-the-art performance in automated DRIL classification.

## 5. Discussion and Analysis

### 5.1. Selecting the Optimal Model and Assessing Performance

The results of cross-validation performance have guided in selecting the optimal model, as the RETFound conservative fine-tuning strategy consistently yielded the highest mean performance for metrics, including AUC (0.9971) and accuracy (0.9733), demonstrating the superior retinal feature extraction power. RETfound exhibited a clear performance improvement when compared with CNN baselines, especially with higher MCC values, reflecting good class balance. Additionally, the results obtained for the held-out test set (AUC: 0.9660, accuracy: 0.9339) by the conservative strategy model indicated a higher ability of the model’s generalization power. This also confirmed that the improvements seen during the cross-validation are not due to overfitting. These consistencies in the results of cross-validation and test evaluations demonstrate the usefulness of selectively finetuning the higher transformer layers while maintaining pretrained representations.

MobileNetV2 results demonstrated that lightweight models can perform better (AUC—0.9858±0.0070 and AC—0.9466±0.0168) despite having fewer parameters than deeper models. These results challenge the notion that medical image classification requires computationally heavy models, indicating that expert-guided anatomical ROI cropping sufficiently eases the classification task for effective models to perform better, and if future validation is performed with external datasets, MobileNetV2 might be used with resource-constrained devices.

### 5.2. Analysis of Fine-Tuning Approaches: Model-Specific Optimization

Important architecture-dependent interactions between fine-tuning methods and model performance were revealed by systematically comparing conservative versus moderate fine-tuning strategies. These findings are crucial for TL optimization in medical imaging applications.

#### 5.2.1. Conservative Fine-Tuning: Retaining Pre-Trained Knowledge

Unfreezing of the top transformer blocks, as in the case of a conservative finetuning strategy, helps in preserving the richer pretrained retinal characteristics, enabling application-specific adaptation. This strategy yielded excellent cross-validation results (AUC: 0.9971 ± 0.0016), representing the fact that minimal changes to the RETFound backbone are sufficient for accurate classification of DRIL. The model seeks to avoid overfitting and maintain stable feature learning (as reflected in the low standard deviation across folds) by limiting the number of layers that are trainable. The ability of the model to generalize is demonstrated through the very good results obtained on the held-out test set (AUC: 0.9660 ± 0.0028). In addition, the high values obtained for specificity and MCC indicate that the model benefits from preserving global contextual features learned through pretraining. Overall, a conservative strategy aims at providing an optimal balance between using pretrained data and adapting to domain-specific features.

#### 5.2.2. Moderate Fine-Tuning-Enabling Deeper Domain Adaptation

The moderate fine-tuning strategy involves unfreezing more layers of transformer blocks, aiming at enhancing deeper domain adaptation for the DRIL detection purpose. Even though the approach yielded strong performance (AUC: 0.9943 ± 0.0035), it did not surpass the conservative approach, implying that excessive parameter updates may not always provide additional benefits. The slight reduction in values for all the performance measures when compared with the conservative approach indicates a possibility of overfitting or reduced generalization when excessive layers are finetuned. This is further reflected in the comparatively higher variability seen across the folds. Moderate fine-tuning provides greater flexibility for learning features; however, the results demonstrate that the pretrained RETFound model already captures most of the retinal features, and thus unfreezing deeper layers yields limited advantages and may lower model stability.

#### 5.2.3. Impact of Learning Rate and Epoch Interaction

In stabilizing the fine-tuning process of the transformer, the interaction between extended training epochs and lower learning rates played a significant role. The learning rates used for the conservative approach ($5\times 10^{-6}$ and $2\times 10^{-6}$) with 40 epochs enabled slower adaptation while preserving the pretrained features. Abrupt updates were mitigated through the use of a warmup cosine scheduler, ensuring smooth convergence. This controlled optimization strategy led to reduced variance and consistent cross-validation outcomes. To achieve optimal performance, the learning rate–epoch dynamics must be carefully tuned.

#### 5.2.4. Impact of Domain-Specific Fine-Tuning in Medical Imaging Applications

The results indicate the usefulness of TL with domain-specific pretrained models like RETFound in retinal OCT classification tasks. RETFound-based learned retinal features helped in achieving superior results with minimal finetuning when compared with the CNN-based models. This demonstrates the significance of pretraining the models with a relevant and enormous amount of data. Improved feature abstraction and class discrimination are shown by the increased AUC and MCC values. Accordingly, in OCT-based analysis, TL greatly improves model efficiency and diagnostic reliability.

### 5.3. Clinical Significance of the Outcomes and the Novel Contribution

DRIL is a potential supporting biomarker associated with visual acuity and disease progression in various retinal diseases like DME. Manual assessment of DRIL is subjective and time-consuming. Automated DRL detection can, therefore, help clinicians in the assessment and monitoring of various eye diseases. Highly reliable detection of DRIL is ensured by higher mean values obtained for sensitivity and specificity measures for the conservatively trained RETFound model. Clinically, this model may support the Ophthalmologists in reducing the manual assessment overhead and may serve as a decision support tool if validated using a few external datasets. The novelty of the proposed work lies in combining transformer-based learning with fovea-centered ROI extraction to enable clinically meaningful and precise feature representation.

The excellent outcomes obtained for the RETFound model indicate our unique method with expert-guided foveal ROI extraction fundamentally enhances automated DRIL detection. Other state-of-the-art approaches that rely on whole OCT B-scan evaluation or computerized foveal identification achieve lower performance even with datasets of the same or larger size. The reason for this suboptimal performance is mostly because these models focused on anatomically irrelevant regions or might have suffered from inaccurate foveal ROI localization due to the presence of pathologies [15,17]. By the use of precise foveal coordinates provided by ophthalmologists with over 23 years of experience, our approach makes sure that the model focuses on the exact 1 mm central region assessed in clinical practice—the region where DRIL has the strongest association with visual acuity and response to the treatment.

### 5.4. Methodological Benefits

The methodology proposed in the research integrates a transformer-based model with a clinically assisted, preprocessed OCT dataset for DRIL classification. The fovea center-based cropping of OCT images ensures that the model concentrates on the exact ROI. The incorporation of fivefold cross-validation with a held-out test set evaluation ensures unbiased result evaluation with robustness. The use of techniques such as label smoothing, TTA, and mixup further helps to enhance the generalization abilities of the model. Additionally, the two fine-tuning approaches used help in flexible optimization depending on the requirements for DRIL classification.

It is evident from the self-attention map interpretability analysis that the model learned morphologically related features, focusing attention on the DRIL regions and the associated retinal layer boundaries, precisely matching the diagnostic criteria specified by the experienced ophthalmologists. This understanding supports regulatory approval for methods requiring explainable AI, increases clinician trust, and makes error analysis easier for continuous progress. The alignment between model-attention-based OCT regions and the clinically significant regions specified by the ophthalmologists suggests that the models have genuinely learned DRIL patterns rather than simply relying on erroneous correlations or image artifacts.

### 5.5. Explainability Analysis

To provide an understanding of the model’s decision-making process, self-attention maps were analyzed for representative examples of correctly classified and misclassified OCT images (Figure 7). Correctly classified DRIL images exhibit more dispersed and irregular attention distributions, corresponding to the presence of DRIL (disrupted retinal layers). In case of the correctly classified No-DRIL cases, attention is mainly concentrated, particularly in areas corresponding to the inner retinal layers, indicating the intactness of layers (the region where DRIL usually appears). In the failure cases, where DRIL images are misclassified as No-DRIL, the attention is seen as diffused or focused on relatively preserved areas, which might cause the model to overlook DRIL presence, resulting in incorrect predictions. These observations indicate that the model predicts between DRIL and normal regions of the OCT images based on variations in the continuity of the retinal layers. However, the attention maps may not always provide sharply localized decisions, and they must be used as qualitative indicators rather than definitive explanations of the model reasoning.

### 5.6. Ablation Study

We performed an ablation study by training the RETFound model using whole OCT B-scans without ROI cropping and under the same 5-fold cross-validation settings. The results are shown in Table 8, and the proposed ROI-guided approach yielded superior AUC (0.9971 vs. 0.9963), specificity (0.9920 vs. 0.9840), and MCC (0.9476 vs. 0.9395) using the conservative approach compared with the whole image approach. This implies that the anatomically supported foveal localization enhances the discriminative feature learning power while eliminating the processing of unnecessary regions in the OCT images.

### 5.7. Limitations and Future Directions

To lower the risk of overfitting, the proposed RETFound model was assessed with five-fold cross-validation on the 85% training dataset and the final model evaluation on an independently held-out 15% test set, which was excluded during training. The conservative strategy yielded consistent cross-validation performance (AUC = 0.9971 ± 0.0016) and also maintained very good performance on the held-out test set (AUC = 0.9660 ± 0.0028), demonstrating good internal generalization and stable learning. The proposed work uses a single-center, single-OCT-device (Zeiss Cirrus HD-OCT 5000) dataset, which may limit the generalizability of the proposed model to other imaging devices and patient groups. Differences in image quality, acquisition protocols, and device characteristics across centers might affect model outcomes. Therefore, the proposed research should be regarded as a proof-of-concept study. To assess the translational robustness of the suggested framework across different real-world clinical settings, additional investigation is necessary. Further validation on multi-center datasets across OCT devices (Heidelberg Spectralis, Zeiss Cirrus, and Topcon), acquisition protocols (varying scan densities and signal strengths), and diverse patient populations (distinct ethnic groups, disease stages, and coexisting conditions) is necessary before clinical deployment. To confirm the real-world clinical generalizability of the model, it is essential to perform external validation on independent datasets from various institutions. As the dataset used in the approach is relatively balanced (429 DRIL, 394 NoDRIL), it may not accurately represent all the real-world scenarios in all the clinical contexts, potentially affecting the validation of models in screening populations with varying prevalence rates.

This research exclusively focuses on the central 1 mm foveal region and is clinically motivated by the fact that the intactness of the concerned region is most important for visual functioning. But this ROI extraction omits the peripheral DRIL extent, which might be relevant for comprehensive DRIL severity assessment, and the process loses the global context of the OCT image. Future work may focus on exploring multi-scale methods to analyze both central and peripheral regions or methods that process the entire 3D OCT volumes to utilize context from adjacent B scans. Further, longitudinal research might be performed to monitor DRIL evolution following anti-VEGF treatment, which could help in predictive modeling of the therapy response and predicting visual outcomes. Incorporation of the longitudinal research may help in prognostic risk stratification beyond binary classification of DRIL using OCT images.

The absence of a quantitative assessment of attention maps against expert-annotated DRIL areas is a limitation of this proposed work that makes it challenging to evaluate the spatial accuracy of the model’s attention. In order to increase clinical interpretability and enable the quantitative evaluation of attention localization, future work will focus on integrating expert annotations for DRIL regions for quantitative validation of the trained model. Furthermore, we recognize as a limitation that validation against multi-center datasets or longitudinal clinical outcomes was not carried out; this will be taken into consideration in subsequent work. The DRIL detection process may also integrate the identification of other DME biomarkers (subretinal fluid, intraretinal cysts, and hyperreflective foci) through multi-task learning models. The development of DL models that can leverage shared representations for overall computerized OCT analysis for the identification of DRIL and other biomarkers seems like a promising area of future research. The incorporation of advanced architectural methods, including different vision transformers (Swin Transformer) and uncertainty quantification through Bayesian deep learning for DRIL detection, may help assess the performance gains and risk stratification.

## 6. Conclusions

This research demonstrated a clinically guided, fovea-centric RETFound-based framework for computerized classification of the DME biomarker, DRIL, in OCT images. The approach integrates domain knowledge with automated assessment by emphasizing the clinically significant central 1 mm foveal region of the OCT images, which is identified through the annotations provided by the expert ophthalmologists. Leveraging 5-fold cross-validation on 85% (699) of training images, the RETFound-based architecture exhibited excellent results across various fine-tuning strategies, achieving consistently high mean AUC values exceeding 0.99, with the conservative strategy demonstrating the highest mean performance. The RETFound model results were further validated on the independent held-out test set (15%), achieving an accuracy of 0.9339±0.0036 and an AUC of 0.9660±0.0028 across the five fold-trained models, demonstrating acceptable generalization to unseen data. The anatomically and clinically supported framework proposed in the study highlights the significance of focusing on the central foveal ROI for precise DRIL detection. EfficientNetB0 and DenseNet121 exhibited stable and superior performance among the pre-trained CNN models, demonstrating their usefulness for OCT DRIL classification. Self-attention map visualizations improved understanding and qualitative assessment of model decision-making by confirming anatomically significant feature learning. The proposed model’s reliability was additionally confirmed by the statistical analysis and ablation studies, which showed statistically consistent performance and validated the significance of anatomically guided fovea-centered ROI selection for precise DRIL classification. Overall, the framework presents promising potential for straightforward and reliable AI-aided DRIL assessment. This study should be regarded as a single-center proof-of-concept and can serve as a foundation for clinically deployable automated DRIL assessment through a combination of expert clinical knowledge integration, if validated further with multi-center and multi-device datasets to ensure robust generalization and interpretable clinical application. In addition to supporting ophthalmologists with routine DRIL identification, this framework provides a useful platform for researchers investigating OCT biomarkers and automated retinal image analysis.

## Figures and Tables

**Table 1:** Summary of DRIL classification studies using OCT images.

(a) Clinical and Observational Studies							
**Reference**	**Dataset Size**					**Agreement Metric**	
Sun et al. [4]	120 eyes					NR	
Radwan et al. [7]	70 eyes (43 DME, 27 non-DME)					κ = 0.88–1.00	
Sun et al. [6]	80 eyes					κ = 0.69–0.77	
Acón & Wu [8]	Review study					NR	
Das et al. [9]	102 eyes (80 patients)					κ = 0.6–1.0	
Joltikov et al. [10]	57 diabetic, 18 controls					*r* = 0.98	
Nakano et al. [11]	37 eyes					CC = 0.93–0.99	
Nadri et al. [12]	104 subjects (78 diabetic)					κ = 0.85	
Di-Luciano et al. [13]	Systematic review (7 studies)					NR	
Singuri et al. [14]	2083 eyes (1175 patients)					κ = 0.88	
Ruiz-Medrano et al. [18]	275 eye					NR	
(**b**) **Deep Learning–Based Studies**							
**Reference**	**Data**	**AC**	**SE**	**SP**	κ	**AUC**	**MCC**
Singh et al. [15]	5992	88.3	82.9	90.0	>0.85	NR	0.70
Tripathi et al. [16]	150	93.3	NR	NR	NR	NR	NR
Toto et al. [17]	442	91.1	91.1	91.1	0.82	0.91	NR
Kodiyalbail Chakrapani et al. [19]	823	99.39	100.0	98.72	0.934	NR	NR

NR: Not reported; CC: Correlation coefficient. AC: Accuracy (%); SE: Sensitivity (%); SP: Specificity (%); AUC: Area under the curve; MCC: Matthews correlation coefficient.

**Table 2:** Description of KMC DRIL-OCT dataset.

Characteristic	Details
Clinical Setting	Single-center dataset acquired at Kasturba Medical College (KMC), Manipal, Karnataka, India
Study Population	Adult male and female patients (>18 years) diagnosed with diabetic macular edema (DME) associated with type 2 diabetes mellitus
Geographical Distribution	Majority of subjects were recruited from KMC Manipal and surrounding regions of Karnataka, India
Number of Subjects	823 unique patients
Number of Eyes	823 (one eye per subject)
Total OCT B-scans	823 (one B-scan per patient)
Class Distribution	DRIL: 429 (52.1%); No-DRIL: 394 (47.9%)
Image Resolution	938 × 625 pixels
Image Format	JPEG
Image Quality Criterion	Only scans with signal strength ≥ 7 were included
OCT Device	Zeiss Cirrus HD OCT 5000
Device Variability	Not evaluated; all scans were acquired using a single OCT platform
Acquisition Protocol	Standard macular cube scanning protocol. Each OCT volume consisted of five raster B-scans, and only the central foveal B-scan was selected.
Annotation Protocol	Independent annotation by two senior ophthalmologists (each with >23 years of clinical experience), followed by consensus reconciliation for discordant cases
Inter-observer Agreement	Overall agreement: 96.7% (796/823); Cohen’s κ=0.934 (95% CI: 0.907–0.961), p<0.001
Ground Truth	Expert consensus labels for DRIL/No-DRIL and foveal center localization

**Table 3:** Interobserver agreement for DRIL classification.

Metric	Value
Total images	823
Overall agreement	96.7% (796/823)
Overall disagreement	3.3% (27/823)
Cohen’s κ (95% CI)	0.934 (0.907–0.961)
*p*-value	<0.001
Interpretation	Excellent
*Observer Classifications*	
Observer 1: DRIL/No-DRIL	425 (51.6%)/398 (48.4%)
Observer 2: DRIL/No-DRIL	420 (51.0%)/403 (49.0%)
*Disagreement Pattern*	
O1 = DRIL, O2 = No-DRIL	16 (1.9%)
O1 = No-DRIL, O2 = DRIL	11 (1.3%)
*Final Consensus*	
DRIL/No-DRIL	429 (52.1%)/394 (47.9%)

CI-confidence interval; O1-Observer 1; O2-Observer 2.

**Table 4:** Cross-validation performance (Mean ± Std Dev) for RETFound under different fine-tuning strategies evaluated on 85% training data. The AUC values additionally report 95% confidence intervals (CI) computed across the five folds.

Strategy	AUC (95% CI)	AC	SE	SP	MCC
Baseline	0.9869±0.0076(0.9802–0.9936)	0.9600±0.0104	0.9455±0.0223	0.9760±0.0261	0.9212±0.0213
Conservative	0.9971±0.0016(0.9957–0.9985)	0.9733±0.0104	0.9564±0.0163	0.9920±0.0179	0.9476±0.0205
Moderate	0.9943±0.0035(0.9912–0.9974)	0.9619±0.0117	0.9418±0.0237	0.9840±0.0219	0.9254±0.0234

**Table 5:** Results on the independent held-out test set (15% of the dataset) obtained using five fold-specific RETFound models trained using cross-validation on the remaining 85% training set (mean ± std dev).

Strategy	AUC	AC	SE	SP	MCC
Conservative	0.9660±0.0028	0.9339±0.0036	0.9262±0.0129	0.9424±0.0093	0.8680±0.0068

**Table 6:** Cross-validation performance (Mean ± Std Dev) for pre-trained models for DRIL-OCT classification.

Model	AUC	AC	SE	SP	MCC
Model 1-MobileNetV2	0.9858±0.0070	0.9466±0.0168	0.9166±0.0240	0.9787±0.0193	0.8953±0.0312
Model 2-EfficientNetB0	0.9875±0.0101	0.9514±0.0191	0.9238±0.0371	0.9822±0.0209	0.9055±0.0363
Model 3-InceptionV3	0.9695±0.0130	0.9235±0.0110	0.9059±0.0367	0.9456±0.0279	0.8497±0.0207
Model 4-DenseNet169	0.9767±0.0119	0.9405±0.0198	0.9019±0.0284	0.9824±0.0103	0.8841±0.0350
Model 5-DenseNet121	0.9850±0.0081	0.9526±0.0203	0.9234±0.0285	0.9847±0.0112	0.9073±0.0388

**Table 7:** Comparison of the proposed method with previously reported deep learning research for OCT-based DRIL classification.

Reference	Images	AC	SE	SP	κ	AUC	MCC	Execution Time (h)
Singh et al. [15]	5992	88.3	82.9	90.0	>0.85	NR	0.70	NR
Tripathi et al. [16]	150	93.3	NR	NR	NR	NR	NR	NR
Toto et al. [17]	442	91.1	91.1	91.1	0.82	91.0	NR	NR
Kodiyalbail Chakrapani et al. [19]	108,309 + 823	99.39	100.0	98.72	0.934	NR	NR	∼50 ^*a*^
Proposed RETFound	823	93.39	92.62	94.24	0.934	96.60	0.8680	1.94 ^*b*^

AC = Accuracy, SE = Sensitivity, SP = Specificity, AUC = Area Under the Curve, MCC = Matthews Correlation Coefficient, NR = Not Reported. Metric values are reported in percentage (%) except Cohen’s κ and MCC. ^*a*^ Approximate execution time corresponding only to self-supervised BYOL backbone pretraining on 108,309 unlabeled OCT images. ^*b*^ Execution time corresponding to the complete RETFound pipeline, including preprocessing, 5-fold cross-validation, fine-tuning, held-out test evaluation, test-time augmentation, and attention map generation.

**Table 8:** Ablation study of fovea-centered ROI guidance using RETFound under different fine-tuning strategies (5-fold cross-validation).

Fine-Tuning	Input	AUC	AC	SE	SP	MCC
Baseline	Whole OCT	0.9932±0.0045	0.9619±0.0165	0.9527±0.0244	0.9720±0.0228	0.9244±0.0326
Baseline	Fovea-centered ROI	0.9869±0.0076	0.9600±0.0104	0.9455±0.0223	0.9760±0.0261	0.9212±0.0213
Conservative	Whole OCT	0.9963±0.0021	0.9695±0.0104	0.9564±0.0163	0.9840±0.0089	0.9395±0.0204
Conservative	Fovea-centered ROI (Proposed)	0.9971±0.0016	0.9733±0.0104	0.9564±0.0163	0.9920±0.0179	0.9476±0.0205
Moderate	Whole OCT	0.9945±0.0036	0.9638±0.0104	0.9418±0.0199	0.9880±0.0110	0.9290±0.0199
Moderate	Fovea-centered ROI	0.9943±0.0035	0.9619±0.0117	0.9418±0.0237	0.9840±0.0219	0.9254±0.0234

## Data Availability

The dataset generated and analyzed during this study is not publicly available due to ethical considerations. However, they can be obtained from the corresponding author upon reasonable request. Code for implementation can be found at GitHub Repository (Version 1.0) https://github.com/ai-research-2025/DRIL-Classification-Benchmark (accessed on 17 May 2026) and Hugging Face (Version 1.0) Demo https://tinyurl.com/Hugging-Face-Demo. (accessed on 17 May 2026).
